# Supplementary material for: Positive Regulatory Roles of Manihot esculenta HAK5 under K+ Deficiency or High Salt Stress
Source: Plants (Basel). 2024 Mar 15;13(6):849. doi: 10.3390/plants13060849 (PMC10974855; doi:10.3390/plants13060849)
Supplement: Supplementary file 1 [file plants-13-00849-s001.zip › Table S1.pdf]

**Table S1. Primer sequences used in the experiment.**	
MeActin-F	TGTTCCCTGGCATTGCAGAC
MeActin-R	CTCGTCGTACTCGCCCTTG
MeHAK5-semiF	TCATGGGCAAAGCTTCGTCG
MeHAK5-semiR	TCGTTGGGGATCAAGCTCAC
MeHAK5-qRT-F	TGAGCCAAGAGAGTACCGGA
MeHAK5-qRT-R	TCCCACTGTTTCAGCGTCTC
MeHAK5-p416-*Bam* HI-F	CGGGATCCGAAGAAGATGGCAGAGGAAGTG
MeHAK5-p416-*Sma* I-R	TCCCCCGGGGCAGCATCATATCTCATATGTC
MeHAK5-GFP-1300-*Sal* I-F	AGCGTCGACATGGCAGAGGAAGTGGGG
MeHAK5-GFP-1300-*Bam* HI-R	CGGGATCCTATCTCATATGTCATTCCGACCC
MeHAK5-1300-*Sal* I-F	AGCGTCGACGAAGAAGATGGCAGAGGAAGTG
MeHAK5-1300-*Bam* HI-R	CGGGATCCGCAGCATCATATCTCATATGTC
