# Supplementary material for: Positive Regulatory Roles of Manihot esculenta HAK5 under K+ Deficiency or High Salt Stress
Source: Plants (Basel). 2024 Mar 15;13(6):849. doi: 10.3390/plants13060849 (PMC10974855; doi:10.3390/plants13060849)
Supplement: Supplementary file 1 [file plants-13-00849-s001.zip › Table S2.pdf]

Table S2. The accession number of HAK5 proteins in various species.	
**Protein name**	**Sequence ID**
MeHAK5	XP_021622847.1
HbHAK5	XP_021678668.2
RcHAK5	XP_015570435.1
PaHAK5	XP_034906503.1
JrHAK5	XP_018829150.1
VvHAK5	RVW65083.1
PvHAK5	XP_031252747.1
TwHAK5	XP_038704437.1
ZmHAK5	NP_001309036.1
OsHAK5	NP_001395942.1
TaHAK5	XP_044354640.1
HvHAK5	XP_044978234.1
GmHAK5	XP_003553810.1
AtHAK5	NP_567404.1
MtHAK5	XP_013457577.1
SbHAK5	XP_021312226.1
