# Supplementary figures and images for: Positive Regulatory Roles of Manihot esculenta HAK5 under K+ Deficiency or High Salt Stress
Source: Plants (Basel). 2024 Mar 15;13(6):849. doi: 10.3390/plants13060849 (PMC10974855; doi:10.3390/plants13060849)

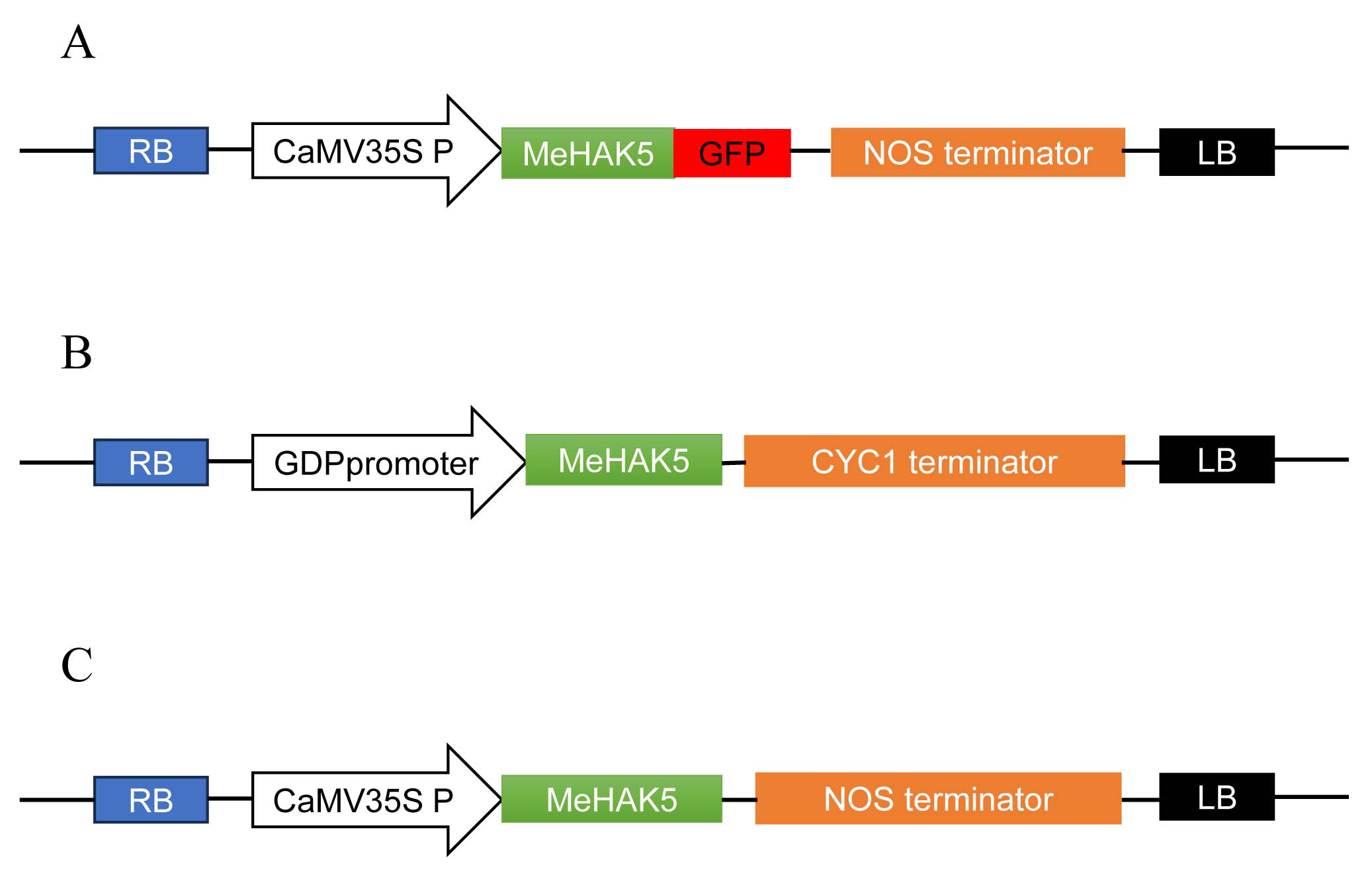

Supplement: Supplementary file 1 [file plants-13-00849-s001.zip › Figure S1.jpg]

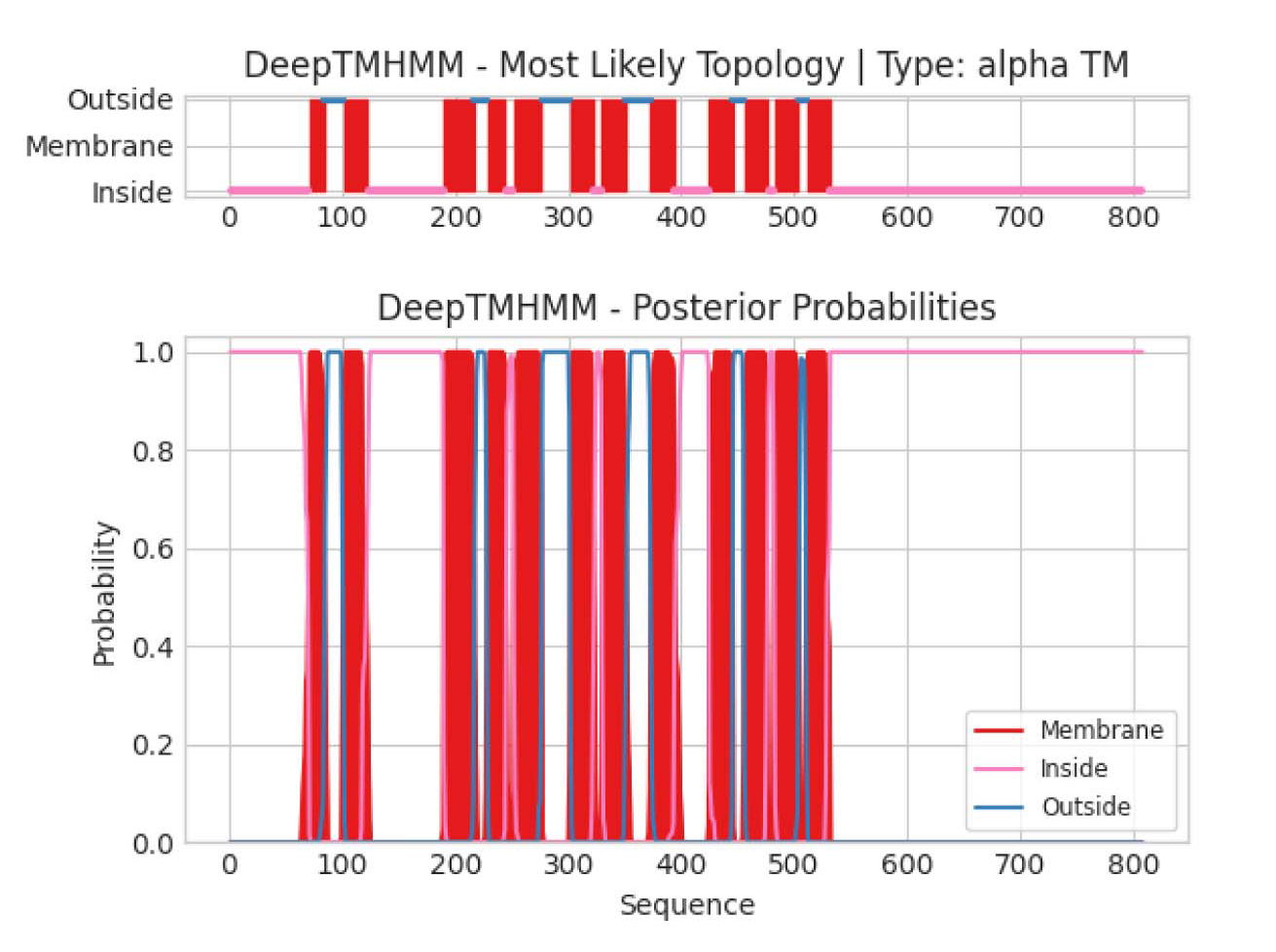

Supplement: Supplementary file 1 [file plants-13-00849-s001.zip › Figure S2.jpg]

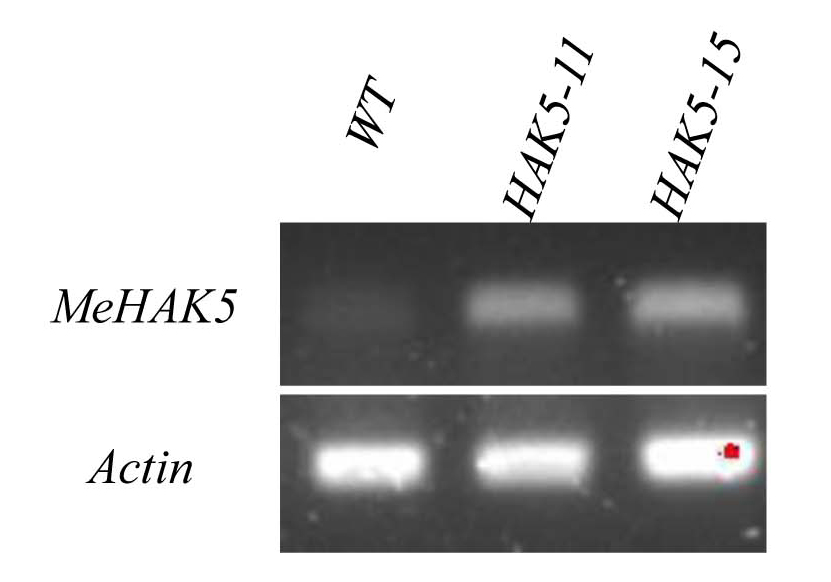

Supplement: Supplementary file 1 [file plants-13-00849-s001.zip › Figure S3.jpg]
